# Thoracic impalement injury by the barrel of a locally fabricated gun in 2 patients: case report

**DOI:** 10.11604/pamj.2022.42.155.33016

**Published:** 2022-06-27

**Authors:** Solomon Ifeanyi Ukwuani, Abubakar Umar, Salisu Ismail, Abdullahi Abdulkarim Aitek, Ibrahim Galadima, Isah Abdullahi, Sulieman Kehinde Ayuba, Ray Bayo

**Affiliations:** 1 Department of Surgery, Cardiothoracic Surgery Unit, Usmanu Danfodiyo University Teaching Hospital, Sokoto, Nigeria,; 2 Department of Anaesthesiology and Intensive Care, Usmanu Danfodiyo University Teaching Hospital, Sokoto, Nigeria

**Keywords:** Impalement, chest injury, gun barrel, case report

## Abstract

Thoracic impalement injuries are uncommon among civilians. When it occurs, it´s usually a severe and dramatic form of chest injury that requires immediate operative intervention. The common mechanisms usually encountered involves either a patient falling from a height onto a pole, being driven into a pole following ejection during a road traffic accident or being impaled when a spear/an arrow is thrown at the patient or from long fragments following a blast. Impalement by a retrograde ejected barrel of a gun during recoil is a very uncommon mechanism. We report 2 recently managed patients. The first patient presented with an overt impaling mass and an initially missed tension pneumothorax. The second patient had a covert impalement chest injury. Both patients had surgical interventions with satisfactory outcomes. Our report aims to highlight this unusual mechanism of thoracic impalement injury and the principles of management. We also want to emphasize the importance of adhering to the advanced trauma life support (ATLS) management algorithm, as immediately life-threatening conditions may be missed when exploratory thoracotomy is the only focus.

## Introduction

Impalement injuries are defined as when large objects or foreign bodies, commonly steel bars or wooden objects, pierce through a body cavity or extremity and remain in place [1]. Thoracic Impalement injuries are uncommon among civilian populations and quite dramatic in presentation [2]. The commonly encountered mechanisms of injury include a fall from a height onto a fixed elongated object, ejection from a car unto a fixed pole during road traffic accidents, and spear/arrow injuries to the chest. Impalement injury from the barrel of a gun is a rare mechanism, with one previously reported by Edwin *et al*. [3]. We present 2 cases of impalement chest injuries from the retrograde ejection of barrels of locally fabricated guns whose housing gave way during discharge, permitting the mechanism of injury. We also want to highlight the principles of management and emphasize the importance of adhering to the advanced trauma life support (ATLS) management algorithm, as immediately life-threatening conditions may be missed when exploratory thoracotomy is the only focus.

## Patient and observation

### Patient 1

**Patient information:** 15-year-old herder with a 3-hour history of worsening difficulty with breathing and chest tightness. He had attempted to discharge a loaded locally fabricated rifle when the barrel recoiled retrograde and impaled him on the chest.

**Figure 1:**
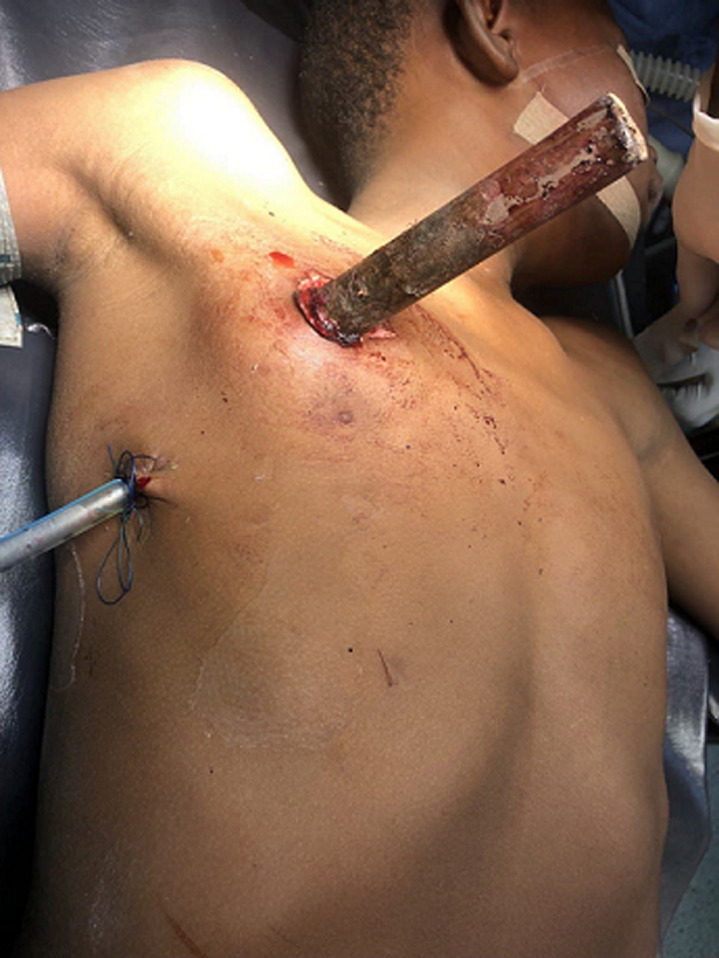
perioperative picture of the first case

**Clinical findings:** he was pale, tachypneic with SPO_2_ of 88% which improved to 94% on supplemental oxygen via nasal prongs. There was a metal pole protruding from the right chest wall, just above the nipple (Figure 1). His blood pressure was 84/51 mmHg with a small, thready pulse of 128 beats per minute. He was commenced on intravenous fluid resuscitation with analgesics following which the cardiothoracic surgeons were invited to review the patient for an exploratory thoracotomy.

**Timeline:** he sustained the chest injury about 3 hours before the presentation, while he was test-firing the firearm used to protect his herd of cattle from rustlers in a rural area about 90 kilometers from the main referral center. He had no prehospital first aid and was transported in a saloon vehicle.

**Diagnostic approach:** subsequent primary survey revealed a bulging right chest hemithorax, hyper resonant percussion note, contralateral tracheal deviation, and almost absent breath sound in the right hemithorax. A clinical diagnosis of tension pneumothorax was made, which was relieved immediately with a chest tube thoracostomy. About 800 mls of blood and a large amount of air were evacuated with remarkable relief of the chest tightness. Imaging investigation was not done because of their unavailability within the trauma center at the time of presentation, and the patient was assessed to be unstable to be moved to the central imaging suite of the hospital. Hemoglobin and renal function tests were within normal limits.

**Figure 2:**
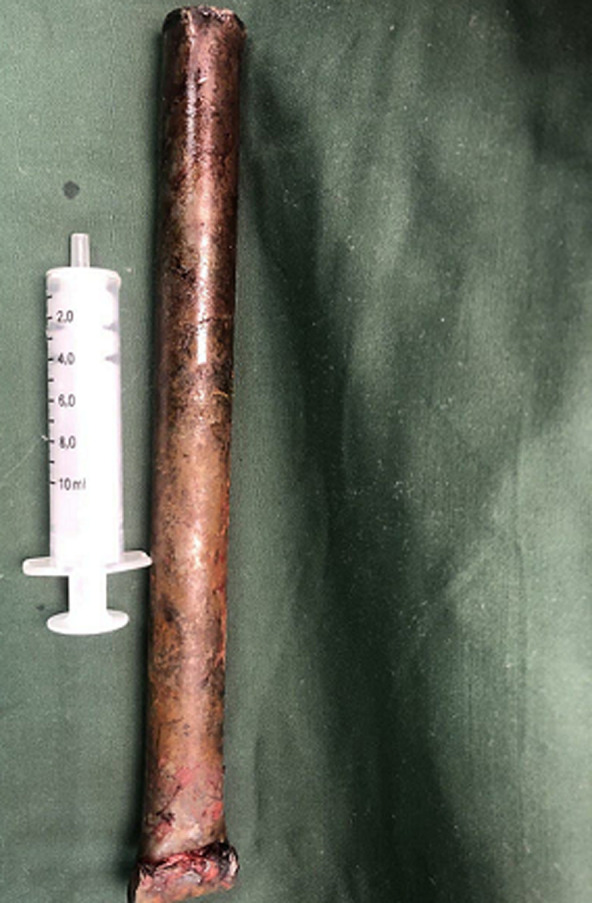
impaling object in the first case

**Therapeutic intervention:** he had an exploratory anterolateral thoracotomy under general anesthesia with controlled retrieval of the pole (Figure 2). Intraoperative findings include injury to the right upper lobe of the lung which was repaired and a comminuted fracture of the 3^rd^ and 4^th^ ribs anteriorly, with the distal end of the pole embedded into the post chest wall. No major vascular injury was encountered. He had broad-spectrum antibiotics and tetanus prophylaxis.

**Follow-up and outcome:** he made a full recovery postoperatively and was discharged after 7 days. Outpatient follow-up visits have been satisfactory.

**Patient´s perspective:** “I´m happy I survived. I never knew the firearm was unsafe. It was supposed to be for my protection.”

**Informed consent:** a written informed consent was obtained from the parents.

### Patient 2

**Patient information:** a 19-year-old vigilante member presented with a day´s history of shortness of breath following impalement injury to the right side of the chest following the retrograde ejection of the recoiling barrel of a locally fabricated rifle he discharged.

**Clinical findings:** he was hemodynamically stable but tachypnoiec. Chest examination revealed a 2 x 3 cm entry wound at about the 3^rd^ right intercostal space, mid-clavicular line, without an obvious impaling object on the anterior chest wall. However, there was an abnormal firm protrusion between the medial border of the scapula and the spine posteriorly.

**Timeline:** sustained the injury a day earlier while trying to fend off armed bandits who had invaded his village in a rural area located about 225 kilometers away. He had no prehospital care.

**Figure 3:**
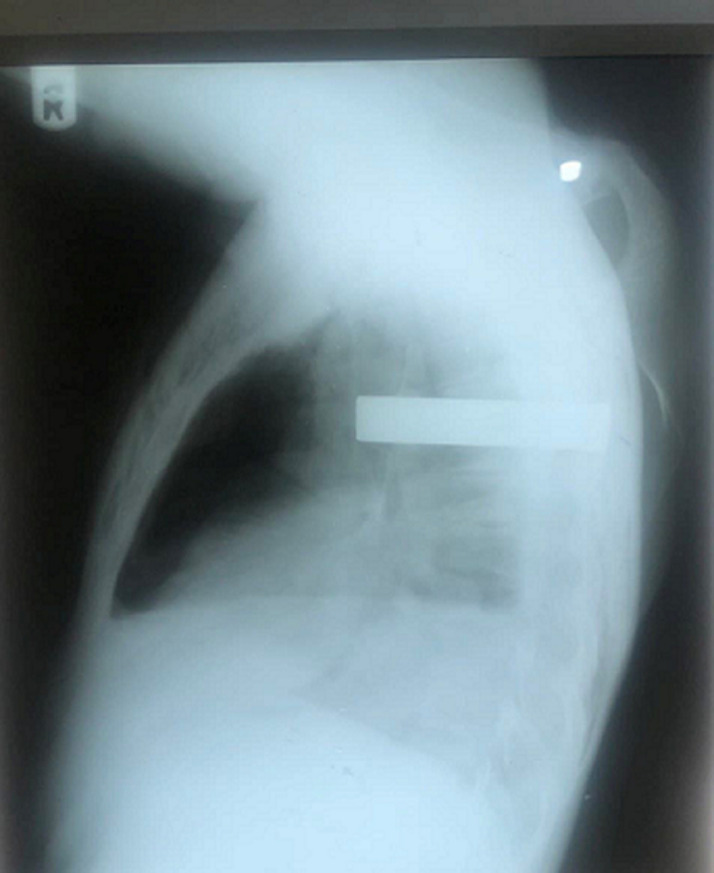
lateral chest radiograph of the second case

**Diagnostic approach:** chest X-ray revealed a radio-opaque cylindrical mass with partial collapse of the right lung (Figure 3). Hemoglobin concentration, white cell count, and renal function test were within normal limits.

**Figure 4:**
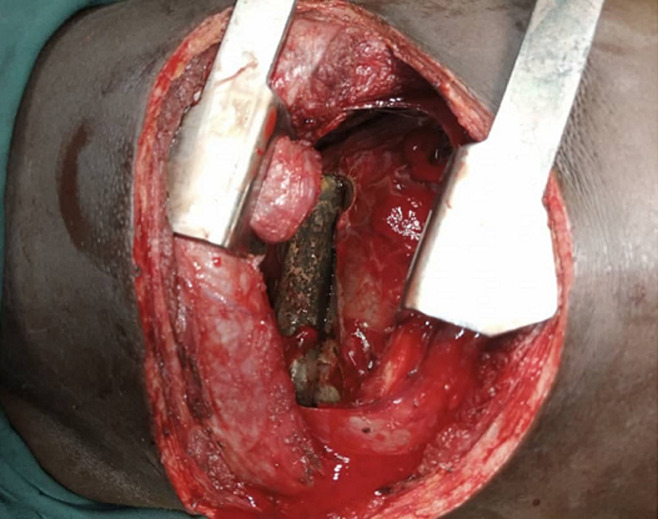
intraoperative picture with the concealed impaling foreign body

**Therapeutic intervention:** he was commenced on antibiotics and tetanus prophylaxis. He had a right exploratory posterolateral thoracotomy with findings of an injured right upper lobe of the lungs, which was repaired and the impaling metal pipe retrieved (Figure 4).

**Follow-up and outcome:** he made a good recovery and was discharged home on the 10^th^ day postoperative.

**Patient´s perspective:** “I was shocked I had this injury. We have a long history of using this type of firearm and such an occurrence has never happened. Thankfully, the metal was safely removed”

**Informed consent:** a written informed consent was obtained from the patient.

## Discussion

Chest trauma is a major contributor to trauma deaths. Although the prevalence of impalement chest injuries is relatively low, many of such patients die before they get to the trauma center due to exsanguinating hemorrhage, especially those involving the central chest and left-sided injuries. Those involving the right side have a better outcome because of the absence of the heart and great vessels [4]. Impalement thoracic injuries are classified based on the etiology/mechanism into [5]. Type I injuries: results from the impact between a moving human body and an immobile object, such as falling on sharp objects, or motor vehicle collisions. These injuries are more common. Type II injuries: occurs as a result of a moving object piercing into an immobile human body, such as in the above patients. This unusual mechanism of injury is explained by Newton´s 3^rd^ law of motion, which states that for every action, there is an equal and opposite reaction. Hence, following the discharge of a firearm, the momentum generated as the bullet/projectile and the exhaust gases travel through the barrel of the gun is balanced by the rearward thrust (recoil) of the gun towards the shooter. With licensed and well-fabricated firearms, the recoiling gun moves backwards as a unit, and this momentum is absorbed by the body of the shooter. However, if the gun housing is defective as is common in locally fabricated guns, the barrel becomes unrestrained following discharge of the firearm and is propelled retrograde towards the shooter causing injuries.

The impaling object is often overt in most cases, as was with our 1^st^ patient. However, it may be covert during the initial evaluation, like in the 2^nd^ patient and other reported cases [3]. As with the general principles of managing impalement injuries, patients should be transported to the hospital with the foreign body insitu, and attempts at removal outside the controlled setting of an operating theater is strongly discouraged, especially when the chest is involved [1,6]. Although the scenarios are often horrifying and distracting spectacular with the first instinct to rush to the operating room, patient initial evaluation should still be structured and based on the Advanced Trauma Life Support (ATLS) algorithm of the American college of surgeons [2,6,7]. This ensures that immediately life-threatening conditions such as a tension pneumothorax are addressed immediately, especially in a resource-limited setting where the time interval between arrival at the trauma center and getting into the operating theater is often not immediate as was the scenario in the first patient and other reported cases [2]. Radiological investigation is not mandatory if not readily available in the vicinity of the trauma center, as moving the patient for such investigation may be counterproductive, especially in hemodynamically unstable patients [1,8]. Since the extent and complexity of injury are often unpredictable, the surgical team should include the most experienced surgeon and anesthetist available [2,9]. A double-lumen endotracheal tube is often helpful and a prophylactic chest tube insertion before the commencement of positive pressure ventilation has been advocated to prevent the development of tension pneumothorax [3]. The surgical access is determined by the expected trajectory of the foreign body and the ease of repair of the likely affected organs and may require unconventional incisions [6]. Because a great deal of energy is required to cause impalement, local tissue destruction can be quite extensive, hence adequate debridement of devitalized tissue and removal of all in-driven clothing must be done with copious lavage of the thoracic cavity. Cardiopulmonary bypass is often required for injuries involving the heart [10]. Broad-spectrum antibiotics and occasionally antifungal agents are mandatory to prevent infectious sequela. For those who survive the prehospital phase and arrive at the trauma center, the chance of survival is often good [1,3,4].

## Conclusion

Thoracic impalement injuries are uncommon forms of severe chest trauma that require immediate and individualized operative care based on the structured algorithm of the ATLS. Although the reported mechanism of injury is unusual, the principles of management are still the same as in all thoracic impalement injuries.
